# Increased Hypothalamic Anti-Inflammatory Mediators in Non-Diabetic Insulin Receptor Substrate 2-Deficient Mice

**DOI:** 10.3390/cells10082085

**Published:** 2021-08-13

**Authors:** María Vinaixa, Sandra Canelles, África González-Murillo, Vítor Ferreira, Diana Grajales, Santiago Guerra-Cantera, Ana Campillo-Calatayud, Manuel Ramírez-Orellana, Óscar Yanes, Laura M. Frago, Ángela M. Valverde, Vicente Barrios

**Affiliations:** 1 Metabolomics Platform, IISPV, Department of Electronic Engineering (DEEEA), Universitat Rovira i Virgili, E-43002 Tarragona, Spain; maria.vinaixa@urv.cat (M.V.); oscar.yanes@urv.cat (Ó.Y.); 2 CIBER de Diabetes y Enfermedades Metabólicas Asociadas (CIBERDEM), Instituto de Salud Carlos III, E-28029 Madrid, Spain; vdasilva@iib.uam.es (V.F.); dgrajales@iib.uam.es (D.G.); 3 Department of Endocrinology, Hospital Infantil Universitario Niño Jesús, Instituto de Investigación La Princesa, E-28009 Madrid, Spain; sandra.canelles@salud.madrid.org (S.C.); santiago.guerra@estudiante.uam.es (S.G.-C.); acampilloc@salud.madrid.org (A.C.-C.); laura.frago@uam.es (L.M.F.); 4 CIBER de Fisiopatología de la Obesidad y Nutrición (CIBEROBN), Instituto de Salud Carlos III, E-28029 Madrid, Spain; 5 Unidad de Terapias Avanzadas, Department of Pediatric Hematology and Oncology, Hospital Infantil Universitario Niño Jesús, Instituto de Investigación La Princesa, E-28009 Madrid, Spain; africa.gonzalez@salud.madrid.org (Á.G.-M.); manuel.ramirez@salud.madrid.org (M.R.-O.); 6 Department of Metabolism and Cell Signaling, Instituto de Investigaciones Biomédicas Alberto Sols (CSIC-UAM), E-28029 Madrid, Spain; 7 Department of Pediatrics, Universidad Autónoma de Madrid, E-28049 Madrid, Spain

**Keywords:** diabetes, hypothalamus, inflammation, IRS2^−/−^ mice, PUFA

## Abstract

Insulin receptor substrate (IRS) 2 is a key mediator of insulin signaling and IRS-2 knockout (IRS2^−/−^) mice are a preclinical model to study the development of diabetes, as they develop peripheral insulin resistance and beta-cell failure. The differential inflammatory profile and insulin signaling in the hypothalamus of non-diabetic (ND) and diabetic (D) IRS2^−/−^ mice might be implicated in the onset of diabetes. Because the lipid profile is related to changes in inflammation and insulin sensitivity, we analyzed whether ND IRS2^−/−^ mice presented a different hypothalamic fatty acid metabolism and lipid pattern than D IRS2^−/−^ mice and the relationship with inflammation and markers of insulin sensitivity. ND IRS2^−/−^ mice showed elevated hypothalamic anti-inflammatory cytokines, while D IRS2^−/−^ mice displayed a proinflammatory profile. The increased activity of enzymes related to the pentose-phosphate route and lipid anabolism and elevated polyunsaturated fatty acid levels were found in the hypothalamus of ND IRS2^−/−^ mice. Conversely, D IRS2^−/−^ mice have no changes in fatty acid composition, but hypothalamic energy balance and markers related to anti-inflammatory and insulin-sensitizing properties were reduced. The data suggest that the concurrence of an anti-inflammatory profile, increased insulin sensitivity and polyunsaturated fatty acids content in the hypothalamus may slow down or delay the onset of diabetes.

## 1. Introduction

Type 2 diabetes is a metabolic disorder that results in insulin resistance and hyperglycemia, and loss of function mutations in the insulin signaling mediators have been related with the onset of the disease [1]. Male insulin receptor substrate (IRS) 2-knockout (IRS2^−/−^) mice are considered a preclinical model of type 2 diabetes because they present hepatic insulin resistance and β-cell failure [2]. Notably, some of these mice present an increase in glycemia comparable to that observed in diabetes onset in humans, whereas a substantial proportion of these mice do not develop diabetes [3,4].

A relationship between changes in insulin signaling and inflammation in metabolic diseases has been investigated [5]. In this regard, the hypothalamus is a target for inflammation where the synthesis of several cytokines has been reported [6]. Hypothalamic microglia is a regulator of metabolic homeostasis, whose dysfunction can result in endocrine diseases [7]. We previously found activation of hypothalamic inflammatory pathways in IRS2^−/−^ mice that distinctively correlate with the presence or absence of diabetes, raising the possibility of an association between inflammation and the onset of diabetes [3].

Insulin promotes glucose uptake to obtain energy for lipogenesis and this hormone modulates the expression of fatty acid synthase (FAS) and stearoyl-CoA desaturase (SCD), the latter catalyzes monounsaturated fatty acid (MUFA) synthesis [8]. Saturated fatty acids induce hypothalamic dysfunction, whereas those unsaturated mediate anti-inflammatory actions [9]. Therefore, changes in lipogenesis and fatty acid profile in the hypothalamus may be associated with modifications in insulin sensitivity [10], central inflammation and diabetes development.

Although inflammation is considered as the primary event in metabolic disturbances, saturated fatty acids may exacerbate this response. In diet-induced obesity models, these diets promote cytokine synthesis and insulin resistance [11], both processes being described in the hypothalamus of diabetic (D) IRS2^−/−^ mice [4]. However, animals fed a monounsaturated fat-rich diet do not develop insulin resistance. This suggests an interconnection among the inflammatory environment, insulin-sensitizing factors, and fatty acid metabolism in the hypothalamus. We hypothesized that in non-diabetic (ND) IRS2^−/−^ mice a favorable pattern of fatty acids in the hypothalamus is associated with a protection against low-grade hypothalamic inflammation. In addition, the differential inflammatory profile in D and ND IRS2^−/−^ mice can be related with modifications in modulating factors of insulin sensitivity [12]. Thus, we determined the pattern of fatty acids, the expression and activity of key enzymes involved in the generation of this profile, as well as a possible association with the inflammatory environment and changes in insulin-sensitizing molecules in the hypothalamus of ND and D IRS2^−/−^ mice. Particularly, we determined different pro-inflammatory (fractalkine, interferon (IFN)-γ, interleukin (IL)-1β, IL-2, IL-17 and monocyte chemoattractant protein (MCP)-1) and anti-inflammatory (IL-4 and IL-13) cytokines given their relationship with insulin sensitivity. We have also measured the activity of enzymes of the pentose phosphate cycle (glucose-6-phosphate dehydrogenase (G6PD) and malic enzyme (ME)) as they generate reducing power for fatty acid synthesis. Moreover, levels and/or activity of lipogenic enzymes (acetyl-CoA carboxylase (ACC), FAS and SCD) were measured in order to explain the changes in hypothalamic levels of fatty acids. In addition, the expression, synthesis and activity of these anabolic enzymes are closely linked to insulin sensitivity. Finally, we determined changes in glucose transporters since they are related to glucose uptake in the hypothalamus that can be used for energy supply, or conversely, for anabolic processes, such as fatty acid synthesis, among others.

## 2. Materials and Methods

### 2.1. Animals

All procedures were carried out in accordance with the local ethics committee and complied with Royal Decree 53/2013 pertaining to the protection of experimental animals and with the European Communities Council Directive (2010/63/EU). The study was conducted according to the guidelines of the Declaration of Helsinki and approved by the Ethics Committee in Animal Experimentation of the Alberto Sols institute (ES 280790000188; 29 October 2018).

Wild-type and IRS2^−/−^ mice, maintained on a similar mixed genetic background (C57BL/6 × 129sv), were previously described [3]. Eighty-two adult, 11- to 12-week-old male mice were fed a standard chow diet (A04, Panlab, Barcelona, Spain) and water ad libitum. Animals were grouped in wild-type (WT) mice, D IRS2^−/−^ mice with non-fasting glucose concentrations over 500 mg/dL, determined by the glucose oxidase method (Precision G glucose meter; Abott Laboratories, North Chicago, IL, USA) and ND IRS2^−/−^ mice with glucose levels under 200 mg/dL. Seven days after the debut of diabetes, animals were sacrificed at 10:00 a.m. under non-fasting conditions. In fifteen animals, the hypothalamus was used for Western blot or bead array assay and in other fifteen mice per group it was used for RNA extraction. In sixteen animals, the hypothalamus was processed for metabolomic studies. In fifteen mice per group, the hypothalamus was isolated and handled for enzymatic activities. In twenty-one animals, the hypothalamus was processed for flow cytometry analysis. Trunk blood was collected in tubes with EDTA and centrifuged at 3000× *g* for 10 min at 4 °C and serum samples were stored at −80 °C.

### 2.2. Antibodies and Reagents

Primary antibodies used were rabbit anti-ACC (C83B10, Cell Signaling Technology, Danvers, MA, USA), rabbit anti-phospho (p)ACC (D7D11, Cell Signaling Technology), mouse anti-actin (1295, Thermo Fisher Scientific, Waltham, MA, USA), mouse anti-AMP-activated protein kinase (AMPK) (2793, Cell Signaling Technology), rabbit anti-pAMPK (2531, Cell Signaling Technology), rabbit anti-glucose transporter (GLUT)1 (GT12-A, Alpha Diagnostic International, San Antonio, TX, USA), goat anti-GLUT2 (sc-31835, Santa Cruz Biotechnology), goat anti-GLUT3 (sc-31840, Santa Cruz Biotechnology, Dallas, TX, USA), mouse anti-GLUT4 (22135, Cell Signaling Technology), mouse anti-FAS (C20G5, Cell Signaling Technology) and mouse anti-vinculin (sc-73614, Santa Cruz Biotechnology). Secondary antibodies used were goat anti-rabbit (P0448, Dako, Denmark), goat anti-mouse (P0447, Dako) and rabbit anti-goat (31402 Invitrogen). All chemicals were from Sigma-Aldrich (St. Louis, MO, USA) unless otherwise noted.

### 2.3. Tissue Homogenization

For immunodetection of ACC, AMPK, FAS, fractalkine, GLUT1–4, IFN-γ, IL-1β, IL-2, -4, -13 and -17, monocyte chemoattractant protein (MCP)-1 and SCD, hypothalamus was homogenized on ice in 400 µL of lysis buffer (Merck, Darmstadt, Germany). The lysates were incubated overnight at −80 °C. Samples were centrifuged at 12,000× *g* for 5 min at 4 °C and the supernatants stored at −80 °C until assayed. For G6PD and ME enzymatic activities, the hypothalamus was homogenized in 400 µL of PBS, following the same protocol and collected in Hanks’ balanced salt solution (HBSS) for flow cytometry analysis. For metabolomic studies, hypothalamic samples were pulverized in liquid nitrogen and processed as described below. Protein concentration was determined by the Bradford method (Bio-Rad Laboratories, Hercules, CA, USA).

### 2.4. Western Blotting

Proteins were separated by electrophoresis on 10% SDS-polyacrylamide gels and incubated with primary antibodies followed with a horseradish peroxidase conjugated secondary antibody. Proteins were detected by chemiluminescence using an ECL system (Bio-Rad Laboratories). Quantification of the bands was carried out by densitometry using a ImageQuant LAS4000 mini TL Software (GE Healthcare Europe GmbH, Munich, Germany). Phosphorylated AMPK and GLUT 1–4 were normalized with Actin, FAS with Vinculin and pACC with its total form.

### 2.5. ELISA

Serum leptin and insulin levels were measured using ELISA kits from Merck according to the manufacturer’s instructions. The determination of serum IGF-I was performed with the OCTEIA immunoenzymometric assay from IDS, Immunodiagnostic Systems Limited (Boldon, Tyne and Wear, UK) following the manufacturer’s instructions. SCD levels were measured using a kit from LifeSpan BioSciences (Seattle, WA, USA). In all cases, the intra- and inter-assay coefficients of variation were lower than 10%.

### 2.6. Enzyme Activity Assays

#### 2.6.1. Glucose-6-Phosphate Dehydrogenase

The activity of this dehydrogenase [EC 1.1.1.49] was assayed with a kit from Sigma-Aldrich, following the manufacturer’s recommendations. After homogenization of the hypothalamus in PBS and subsequent centrifugation, supernatants were incubated at 37 °C with master reaction mix and absorbance at 450 nm measured.

#### 2.6.2. Malic Enzyme

The activity of this enzyme [EC 1.1.1.40] was determined according to the method of Geer et al. [13]. Diluted supernatants were incubated at 25 °C with a triethanolamine buffer, malic acid and the NADP and absorbance was monitored at 340 nm.

### 2.7. Metabolomics

#### 2.7.1. Brain Extraction

Protocol for metabolites brain extraction was partly adapted from Shi et al. [14]. Powdered hypothalamic tissue (~100 mg) was homogenized for 5 min at 0 °C in 2 mL of a cold mixture (acetonitrile/water, 1:1 *v*/*v*). The suspension was centrifuged at 4 °C for 15 min at 5000× *g* and the supernatant containing hydrophilic extracted metabolites was transferred into a new tube. This step was repeated three times and the aqueous phases were frozen, lyophilized and kept at −80 °C. Pellets containing lipophilic-soluble metabolites were dried under a N_2_ stream and shaken in a 1 mL solution (chloroform/methanol, 3:1 *v/v*) during 20 min. The mixture was centrifuged at 4 °C for 30 min at 22,600× *g* and the supernatant was dried under N_2_ stream and stored at −80 °C until nuclear magnetic resonance (NMR) analysis.

#### 2.7.2. NMR Measurements

The hydrophilic extracts were reconstituted in 600 µL of D_2_O phosphate buffer (PBS 0.05 mM, pH 7.4, 99.5% D_2_O) containing 0.58 mM trisilylpropionic acid (TSP) and the solution was transferred into a 5-mm NMR glass tube. 1D-NOESY ^1^H-NMR spectra were recorded at 300 K on an Avance III 600 spectrometer (Bruker, Bremen, Germany) operating at a proton frequency of 600.20 MHz and equipped with an inverse TCI 5 mm cryoprobe. Solvent presaturation with low irradiation power (10 Hz) was applied during recycling delay (RD = 8 s) and mixing time (tm = 100 ms) to suppress residual water. Lipophilic extracts were reconstituted into a deuterochloroform/tetradeuteromethanol (2:1 *v/v*) solution containing 0.73 mM tetramethylsilane (TMS) and a 90° pulse with a presaturation sequence (zgpr) was used to measure corresponding 1D-NMR spectra. We performed measurements at 287 K, shifting the residual water signal to 4.65 ppm to allow for the quantification of the characteristic glycerol-backbone signals. A total of 256 transients were collected across 12 kHz (20 ppm) spectral width at 300 K into 64 K data points, and an exponential line broadening of 0.3 Hz was applied before Fourier transformation. 

#### 2.7.3. NMR Data Analysis

The frequency domain spectra were phased, baseline-corrected, and referenced to TSP or TMS signal (δ = 0 ppm) using TopSpin software (version 2.1, Bruker). Resonance assignments were done based on literature values [15] and different database search engines (Bioref AMIX 3.8 database from Bruker, Billerica, MA, USA), Chenomx NMRSuite 7.5 from Chenomx Inc. (Edmonton, AB, Canada) and Human Metabolome Database, version 3.0 (HMDB, http://www.hmdb.ca, accessed on 9 November 2015). Structural confirmations were based on two-dimensional (2D) ^1^H-^1^H COrrelation SpectroscopY (COSY) and 2D ^1^H-^13^C Heteronuclear Single Quantum Correlation (HSQC). Resonances were integrated using AMIX 3.9 software package (Bruker).

### 2.8. Multiplexed Bead Immunoassay

Plasma and hypothalamic levels of fractalkine, IFN-γ, IL-1β, IL-2, IL-4, IL-13, IL-17 and MCP-1 were measured by multiplexed bead immunoassay (Merck) [16]. Beads conjugated to the antibodies and tissue lysates were incubated for 18 h. Afterwards, wells were washed, and antibody conjugated to biotin was added. After incubation for 30 min, beads were incubated with streptavidin-phycoerythrin. A minimum of 50 beads per parameter were analyzed in the Bio-Plex suspension array system 200 (Bio-Rad Laboratories). Raw data (median fluorescence intensity,) were analyzed with the Bio-Plex Manager Software 4.1 (Bio-Rad Laboratories). Coefficients of variation were lower than 10%.

### 2.9. Flow Cytometry Analysis

Blood was collected in tubes containing EDTA. Once the hypothalamus was collected in HBSS, tissue was disaggregated and filtered in a membrane of 40 µm. Next, the labeling of the surface antigens was carried out for 30 min at 4 °C. The surface antibodies and their fluorescences used were: CD206-FITC, CD11-PE, F4/80-PECY7, CD11B-APC, Ly6G-APC-CY7, Ly6C-PacificBlue and CD45-V500 (Biolegend, San Diego, CA, USA). To distinguish between living and dead cells we used 7-aminoactinomycin labeling (Biolegend). After labeling, samples were incubated for 20 min with Quick Lysis buffer (Cytognos, Salamanca, Spain). Samples were analyzed in a BD FACSCanto II flow cytometer (Becton Dickinson, New York, NY, USA) using FACSDiva software. The flow cytometer settings and samples were prepared according to the manufacturer’s instructions. The intensity of surface expression was measured as MFI. The positive stained cells were expressed as percentage values.

### 2.10. RNA Extraction, Reverse Transcription, and Real-Time PCR

Total RNA was extracted according to the Tri-Reagent protocol (Sigma-Aldrich). The reverse transcription reaction was performed on 2 μg of total RNA using the high-capacity cDNA archive kit (Thermo Fisher Scientific, Waltham, MA, USA). Real-time PCR was performed in an ABI Prism 7000 Sequence Detection System using TaqMan PCR Master Mix (Thermo Fisher Scientific) and the thermocycler parameters recommended by the manufacturer. PCRs were performed in a total volume of 50 μL, containing 12.5 μL of the reverse transcription reaction. TaqMan gene expression assays were used for the analysis of mRNAs encoding carnitine palmitoyl transferase (CPT)1a, lipoprotein lipase (LPL) and phosphoglycerate kinase (PGK)1 (Mm01231183_m1 Mm00434764_m1 and Mm00435617_m1, respectively; Thermo Fisher Scientific). mRNAs encoding for arginase (Arg)1, mannose receptor C (MCR)1 and macrophage galactose-C type lectin (MGL)1 were measured with SYBR Green (Roche, Mannheim, Germany), with primers purchased from Sigma-Aldrich. The PCR mixture contained 300 nM of each primer. Relative gene expression comparisons were carried out using an invariant endogenous control (GAPDH). The ΔΔCT method was used for relative quantification.

### 2.11. Statistical Analysis

Data are summarized as mean ± SEM. Statistical analysis of all data was carried out by one-way ANOVA followed by a Bonferroni’s test. Relationships between variables were performed by linear regression analysis. *p* < 0.05 was considered significant. Analyses were performed using Statview software (Statview 5.01, SAS Institute, Cary, NC, USA) and graphs were made by GraphPad Prism 8 software (San Diego, CA, USA).

## 3. Results

### 3.1. Serum Glucose and Hormones in IRS2^−/−^ Mice

Glycemia was increased in both IRS2^−/−^ mice, being more elevated in D IRS2^−/−^ mice. Insulin levels were augmented in ND and D IRS2^−/−^ mice; this increase being higher in ND IRS2^−/−^ mice. HOMA-IR index was augmented in both IRS2^−/−^ mice but with a major impact in D IRS2^−/−^ mice. Serum leptin was diminished in D IRS2^−/−^ mice and increased in ND IRS2^−/−^ mice. Peripheral IGF-I levels were increased in ND IRS2^−/−^ mice with respect to WT and D IRS2^−/−^ mice (Table 1).

### 3.2. Glucose Metabolism and Energy Status in IRS2^−/−^ Mice

**Figure 1:**
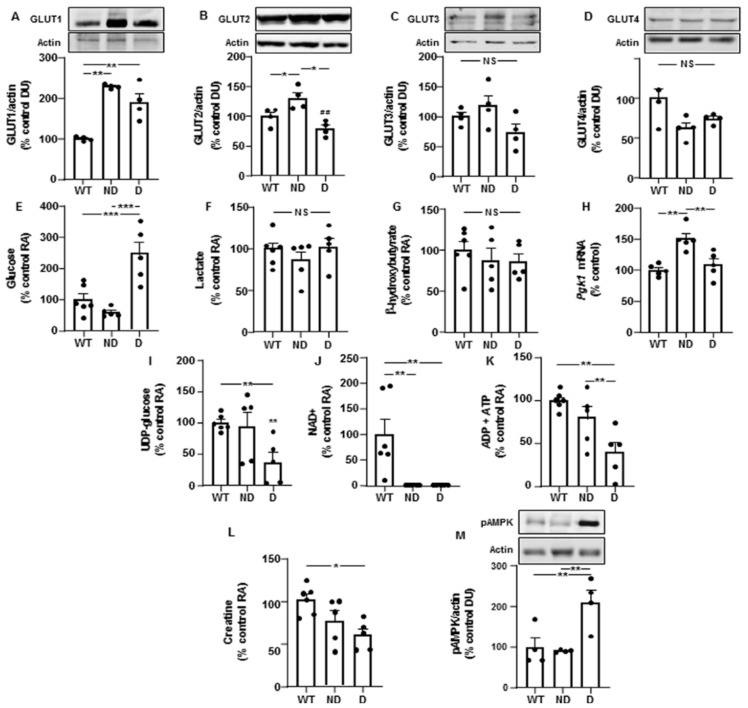
Changes in the levels of glucose transporters, metabolites and energy status in the hypothalamus of IRS2^−/−^ mice. (**A**) Relative protein levels of GLUT-1 in WT, ND IRS2^−/−^ and D IRS2^−/−^ mice. (**B**) Relative protein levels of GLUT-2. (**C**) Relative protein levels of GLUT-3. (**D**) Relative protein levels of GLUT-4. (**E**) Relative levels of glucose. (**F**) Relative levels of lactate. (**G**) Relative levels of β-hydroxybutyrate. (**H**) Relative phosphoglycerate kinase 1 (*Pgk-1*) mRNA levels. (**I**) Relative levels of UDP (uridine diphosphate)-glucose. (**J**) Relative levels of nicotinamide adenine dinucleotide (NAD^+^). (**K**) Relative levels of ADP plus ATP. (**L**) Relative levels of creatine. (**M**) Relative phosphorylated (*p*) levels of AMP-activated protein kinase (pAMPK). Data are presented as means ± SEM. DU, densitometry units; NS, non-significant; RA, relative area. * *p* < 0.05, ** *p* < 0.01, *** *p* < 0.001 by ANOVA.

We first studied protein levels of glucose transporters-1 to -4 in the hypothalamus, where they have been previously described [17]. Levels of GLUT-1 were augmented in both ND and D IRS2^−/−^ mice compared to those of WT controls (Figure 1A). GLUT-2 was increased in ND IRS2^−/−^ mice compared to WT or D IRS2^−/−^ mice (Figure 1B). Levels of GLUT-3 and GLUT-4 were not different among the experimental groups (Figure 1C,D, respectively).

Hypothalamic glucose levels were increased in D IRS2^−/−^ mice compared to WT or ND IRS2^−/−^ mice (Figure 1E). No differences in lactate and β-hydroxybutyrate levels were found (Figure 1F,G, respectively). We found higher *Pgk1* mRNA levels in ND IRS2^−/−^ mice than in WT or D IRS2^−/−^ mice (Figure 1H). D IRS2^−/−^ mice presented lower levels of uridine diphosphate (UDP)-glucose than WT and ND IRS2^−/−^ mice (Figure 1I).

NAD^+^ levels were depleted in both ND and D IRS2^−/−^ mice (Figure 1J) and levels of ADP plus ATP were reduced in D IRS2^−/−^ mice compared to WT and ND IRS2^−/−^ mice (Figure 1K). Creatine was diminished in D IRS2^−/−^ mice (Figure 1L) and activation of AMPK was increased in D IRS2^−/−^ mice (Figure 1M).

### 3.3. Unsaturated Fatty Acids and Lipid Anabolism-Related Enzymes Are Increased in ND IRS2^−/−^ Mice

**Figure 2:**
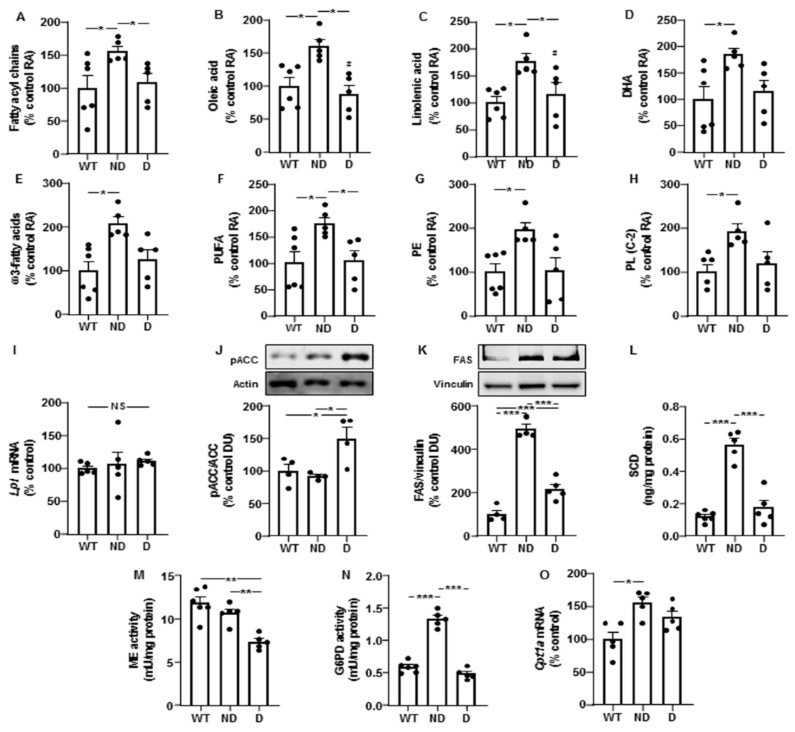
Lipid profile and changes in enzymes of lipid metabolism in the hypothalamus of IRS2^−/−^ mice. (**A**) Relative levels of fatty acyl chains in WT, ND IRS2^−/−^ and D IRS2^−/−^ mice. (**B**) Relative levels of oleic acid. (**C**) Relative levels of linolenic acid. (**D**) Relative levels of docosahexaenoic acid (DHA). (**E**) Relative levels of ω3-fatty acids. (**F**) Relative levels of polyunsaturated fatty acids (PUFA). (**G**) Relative levels of phosphatidylethanolamine (PE). (**H**) Relative levels of phospholipids (PL) C-2. (I) Relative lipoprotein lipase (*Lpl*) mRNA content. (**J**) Relative phosphorylated (*p*) levels of acetyl-CoA carboxylase (pACC). (**K**) Relative protein levels of fatty acid synthase (FAS). (**L**) Protein concentrations of stearoyl-CoA desaturase (SCD). (**M**) Malic enzyme (ME) activity. (**N**) Glucose-6-phosphate dehydrogenase (G6PD) activity. (**O**) Relative carnitine palmitoyltransferase 1a (*Cpt-1a*) mRNA content. Data are presented as means ± SEM. DU, densitometry units; NS, non-significant; RA, relative area. * *p* < 0.05, ** *p* < 0.01, *** *p* < 0.001 by ANOVA.

Hypothalamic levels of fatty acyl chains and oleic acid were increased in ND IRS2^−/−^ mice compared to those presented by WT or D IRS2^−/−^ mice (Figure 2A,B, respectively).

Levels of linoleic acid did not change (100 ± 19, 169 ± 30 and 117 ± 27, expressed in percentage of control values in WT, ND and D IRS2^−/−^ mice, respectively). Linolenic acid was augmented in ND IRS2^−/−^ mice with respect to WT or D IRS2^−/−^ mice (Figure 2C) and docosahexaenoic acid (DHA) increased in ND IRS2^−/−^ mice (Figure 2D). ND IRS2^−/−^ mice presented higher levels of ω3-fatty acids and polyunsaturated fatty acids (PUFA, Figure 2E,F, respectively). Phosphatidylethanolamine (PE) and total phospholipids C-2 were augmented in ND IRS2^−/−^ mice (Figure 2G,H, respectively).

We did not find differences in *Lpl* mRNA levels (Figure 2I). Phosphorylation of ACC in serine residues inhibits its enzymatic activity [18]. We found an increase in ACC phosphorylation in D IRS2^−/−^ mice (Figure 2J). ND and D IRS2^−/−^ mice had elevations in FAS protein content, with a higher effect in ND IRS2^−/−^ mice (Figure 2K). SCD protein levels in ND IRS2^−/−^ mice were increased with respect to WT and D IRS2^−/−^ mice (Figure 2L).

We analyzed ME and G6PD activities, both involved in the generation of the reduced form of nicotinamide adenine dinucleotide phosphate (NADPH) for lipogenesis. Whereas the activity of ME was diminished in D IRS2^−/−^ mice (Figure 2M), G6PD activity increased in ND IRS2^−/−^ mice (Figure 2N). To determine possible changes in fatty acid catabolism in ND and D IRS2^−/−^ mice, we measured the expression of carnitine palmitoyltransferase 1a (*Cpt1a*). As shown in Figure 2O, *Cpt1a* mRNA levels were augmented in ND IRS2^−/−^ mice.

### 3.4. Proinflammatory and Anti-Inflammatory Markers in Plasma and Hypothalamus of ND and D IRS2^−/−^ Mice

**Figure 3:**
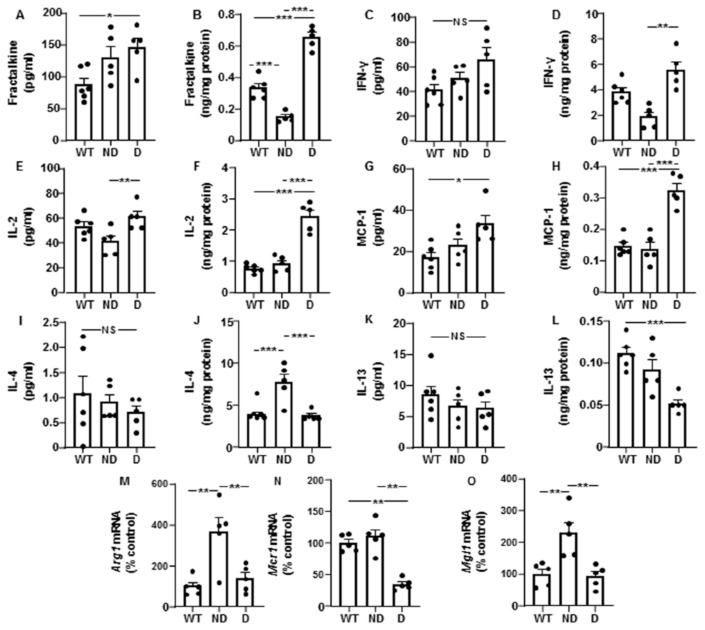
Cytokine levels in serum and hypothalamus and hypothalamic anti-inflammatory markers in IRS2^−/−^ mice. (**A**) Plasma fractalkine concentrations in WT, ND IRS2^−/−^ and D IRS2^−/−^ mice. (**B**) Hypothalamic fractalkine levels. (**C**) Plasma interferon (IFN)-γ concentrations. (**D**) Hypothalamic IFN-γ levels. (**E**) Plasma interleukin (IL)-2 concentrations. (**F**) Hypothalamic IL-2 levels. (**G**) Plasma monocyte chemoattractant protein (MCP)-1 concentrations. (**H**) Hypothalamic MCP-1 levels. (**I**) Plasma IL-4 concentrations. (**J**) Hypothalamic IL-4 levels. (**K**) Plasma IL-13 concentrations. (**L**) Hypothalamic IL-13 levels. (**M**) Relative arginase 1 (*Arg-1*) mRNA content in the hypothalamus. (**N**) Relative mannose receptor C 1 (*Mcr-1*) mRNA content in the hypothalamus. (**O**) Relative macrophage galactose-C type lectin 1 (*Mgl-1*) mRNA levels in the hypothalamus. Data are presented as means ± SEM. NS, non-significant. * *p* < 0.05, ** *p* < 0.01, *** *p* < 0.001 by ANOVA.

Plasma fractalkine was increased in D IRS2^−/−^ mice and hypothalamic levels were reduced in ND IRS2^−^ mice and increased in D IRS2^−/−^ mice (Figure 3A,B, respectively). No changes in plasma IFN-γ were found; however, its hypothalamic levels were increased in D IRS2^−/−^ mice (Figure 3C,D, respectively). Circulating IL-2 was augmented in D IRS2^−/−^ mice with respect to ND IRS2^−/−^ mice and hypothalamic concentrations were increased in D IRS2^−/−^ mice compared to WT or ND IRS2^−/−^ mice (Figure 3E,F, respectively). Circulating and hypothalamic MCP-1 levels were augmented in D IRS2^−/−^ mice (Figure 3G,H, respectively).

We also measured other pro-inflammatory IL. Plasma IL-1β levels were augmented in D IRS2^−/−^ mice and hypothalamic concentrations were reduced in ND IRS2^−/−^ mice while increased in D IRS2^−/−^ mice. Peripheral and hypothalamic IL-17 levels were increased in D IRS2^−/−^ mice but not in in ND IRS2^−/−^ mice (Table 1).

We also analyzed anti-inflammatory IL. There were no differences in the circulating concentrations of IL-4; however, its protein levels were increased in the hypothalamus of ND IRS2^−/−^ mice (Figure 3I,J, respectively). Circulating IL-13 levels were similar among the experimental groups but its hypothalamic expression was reduced in D IRS2^−/−^ mice (Figure 3K,L, respectively). Notably, hypothalamic *Arg1* expression was increased in ND IRS2^−/−^ mice (Figure 3M) and *Mcr1* mRNA levels were reduced in D IRS2^−/−^ mice (Figure 3N). *Mgl1* mRNA levels were up-regulated in ND IRS2^−/−^ mice (Figure 3O).

**Figure 4:**
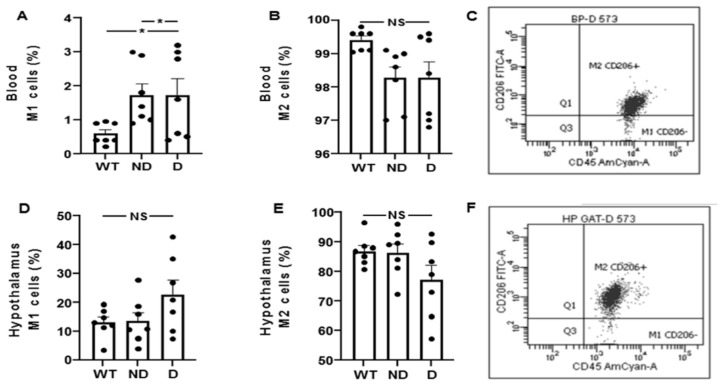
Analysis of M1 and M2 cells by flow cytometry in blood and hypothalamus of IRS2^−/−^ mice. (**A**) Percentage of M1 cells (CD11b^+^/CD45^+^/CD206^-^) in blood of WT, ND IRS2^−/−^ and D IRS2^−/−^ mice. (**B**) Percentage of M2 (CD11b^+^/CD45^+^/CD206^+^) cells in blood. (**C**) Flow cytometry strategy of analysis and quantification of macrophages. (**D**) Percentage of cells with M1 phenotype in hypothalamus. (**E**) Percentage of cells with M2 phenotype in hypothalamus. (**F**) Flow cytometry strategy of analysis and quantification of microglia cells. Because myeloid CD11b^+^ cells are present in a very low percentage in hypothalamus (around 0.2%), we acquired the gated CD11b^+^ population. Macrophages status from blood in M1 is CD45^high^CD11b^+^Ly6G^low^Ly6c^low^CD206^−^ and M2 status is CD45^high^CD11b^+^Ly6G^low^Ly6c^low^CD206^+^. Microglia status from hypothalamus in M1 is CD45^low^CD11b^+^Ly6G^low^Ly6c^low^CD206^−^ and M2 status is CD45^low^CD11b^+^Ly6G^low^Ly6c^low^ CD206^+^. Data are presented as means ± SEM. NS, non-significant. * *p* < 0.05 by ANOVA.

Next, we characterized the M1 and M2 phenotype attending to the expression of the CD206 marker in macrophages (CD11b + CD45high) and microglia (CD11b + CD45low) populations in WT, D and ND IRS2^−/−^ mice. The percentage of blood M1 cells was increased in ND and D IRS2^−/−^ mice (ND: 393%; D: 378% of WT values, Figure 4A). However, no differences in blood M2 cells were found (ND: 98%; D: 99% of WT values, Figure 4B). Flow cytometry and quantification is shown in Figure 4C. The percentage of hypothalamic cells with M1 phenotype was similar in the experimental groups (ND: 104%; D: 170% of WT values, Figure 4D). Likewise, the percentage of M2 cells in the hypothalamus did not show differences (ND: 99%; D: 90% of WT values, Figure 4E). Flow cytometry and quantification is shown in Figure 4F.

### 3.5. Molecules Associated with Insulin-Sensitizing and Anti-Inflammatory Effects in ND and D IRS^−/−^ Mice

**Figure 5:**
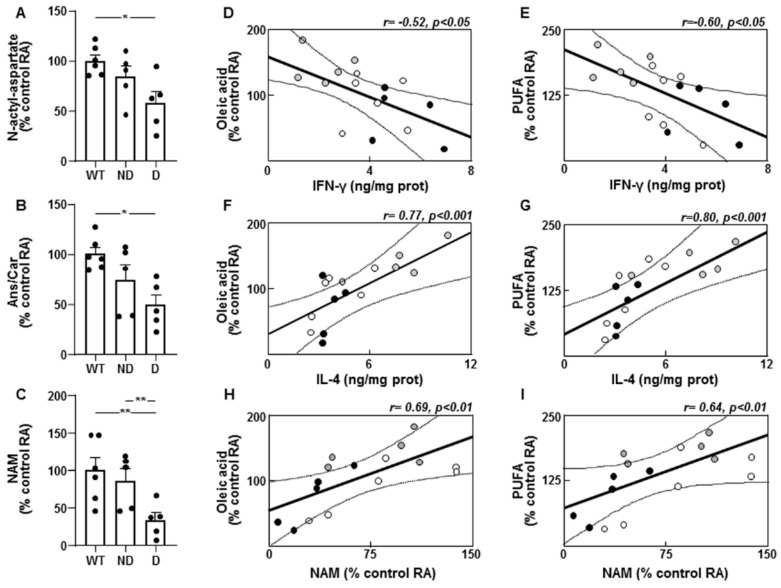
Molecules related to an anti-inflammatory environment and correlations between unsaturated fatty acids and pro- and anti-inflammatory markers in the hypothalamus of IRS2^−/−^ mice. (**A**) Relative levels of N-acetyl aspartate in WT, ND IRS2^−/−^ and D IRS2^−/−^ mice. (**B**) Relative levels of anserine/carnosine (Ans/Car). (**C**) Relative levels of niacinamide (NAM). (**D**) Linear correlation between oleic acid and interferon (IFN)-γ concentrations. (**E**) Linear correlation between polyunsaturated fatty acids (PUFA) and IFN-γ concentrations in WT (white circle), ND IRS2^−/−^ (grey circle) and D IRS2^−/−^ mice (black circle). (**F**) Linear correlation between oleic acid and interleukin (IL)-4 concentrations. (**G**) Linear correlation between PUFA and IL-4 concentrations. (**H**) Linear correlation between oleic acid and NAM concentrations. (**I**) Linear correlation between PUFA and NAM concentrations. Data are presented as means ± SEM. Correlation coefficients (r) and *p* values are represented for each analysis. RA, relative area. * *p* < 0.05, ** *p* < 0.01 by ANOVA.

Levels of N-acetyl aspartate were lower in the hypothalamus of D IRS2^−/−^ mice (Figure 5A) and levels of carnosine and its methylated form, anserine, were decreased in D IRS2^−/−^ mice (Figure 5B). Niacinamide (NAM) presented lower concentrations in D IRS2^−/−^ mice with respect to WT or ND IRS2^−/−^ mice (Figure 5C).

### 3.6. Correlation between Cytokines with Unsaturated Fatty Acid Levels and Enzymes Involved in Lipid Metabolism in the Hypothalamus

Oleic acid or PUFA levels were inversely correlated with IFN-γ (Figure 5D,E, respectively). These fatty acids showed a direct correlation with IL-4 (Figure 5F,G, respectively) and NAM (Figure 5H,I, respectively).

Fractalkine showed a negative correlation with oleic acid (r = −0.51, *p* < 0.05). This cytokine and IFN-γ had an inverse relationship with SCD levels (r = −0.55, *p* < 0.05 and r = −0.69, *p* < 0.001, respectively) and ME activity (r = −0.59, *p* < 0.01 and r = −0.52, *p* < 0.05, respectively). Hypothalamic IL-4 levels were directly correlated with FAS (r = 0.90, *p* < 0.001) and SCD levels (r = 0.91, *p* < 0.001) as well as G6PD activity (r = 0.66, *p* < 0.001).

No correlations were found between serum levels of IFN-γ, IL-4 or fractalkine and unsaturated fatty acid levels or lipogenic enzymes in the hypothalamus (data not shown).

### 3.7. Correlation between Serum Insulin Levels with Hypothalamic Enzymes, Fatty Acids and Anti-Inflammatory Cytokines

We also determined the relationship between serum insulin levels, hypothalamic pentose-phosphate route and lipid anabolism-related enzymes, polyunsaturated fatty acid levels and anti-inflammatory IL. We found a direct correlation of insulin with G6PD (r = 0.82, *p* < 0.001), FAS (r = 0.87, *p* < 0.001), PUFA (r = 0.61, *p* < 0.01) and IL-4 (r = 0.83, *p* < 0.001).

## 4. Discussion

Hypothalamic fatty acid metabolism is key in transmitting the central action of fatty acids on energy homeostasis; the latter associated with insulin signaling and changes in inflammation. Here, we report elevations in mono- and poly-unsaturated fatty acids (MUFAs and PUFAs, respectively) in ND IRS2^−/−^ mice related to an increase in lipogenic enzymes, whereas D IRS2^−/−^ mice presented a reduction in molecules associated with anti-inflammatory and insulin-sensitizing effects. We also provide evidence that hypothalamic and systemic inflammation were increased in D IRS2^−/−^ mice, while ND IRS2^−/−^ mice presented an increase in hypothalamic anti-inflammatory cytokines; being more relevant than their levels in circulation.

An adequate uptake of glucose and a high rate of glycolytic flux is necessary for ATP production in the brain. We found an increase in GLUT2 in ND IRS2^−/−^ mice, an essential transporter for glucose uptake in the hypothalamus [20]. Inflammation may diminish GLUT2, whereas IL-4 enhances glucose uptake by upregulating GLUT2 expression and increasing insulin-induced glucose uptake [21]. Diabetic IRS2^−/−^ mice presented an increase in hypothalamic glucose levels possibly due to its abnormal utilization, as suggested by the reduced UDP-glucose levels, whereas ND IRS2^−/−^ mice showed an increased expression of *Pgk1*, a key enzyme in glycolysis, whose activity is stimulated by insulin [22]. Experimental activation of AKT in type 2 diabetic mice restores glycogen synthesis and inhibits gluconeogenesis [23], whereas most of the inflammatory states are associated with reduced glycogen synthesis [24].

Hyperglycemia and diabetes are associated with an upregulation of gluconeogenic enzymes, while factors involved in oxidative phosphorylation are downregulated. Thus, D IRS2^−/−^ mice presented low levels of ATP and creatine, whereas ND IRS2^−/−^ mice showed values comparable to WT mice, suggesting an adequate energy status. Creatine plays a key role as buffering energy supply in tissues with high-energy demand, such as the brain [25]. However, both D and ND IRS2^−/−^ mice have extremely low levels of NAD. This result is somehow surprising, but our data in ND IRS2^−/−^ mice suggest utilization of the obtained energy for anabolic processes.

AMPK is activated in situations of inadequate energy balance and our results showed an increase in D IRS2^−/−^ mice that presented hyperphagia [3]. AMPK activation increases food intake and is responsible for inactivating ACC [26], a rate-limiting step in fatty acid biosynthesis. ND IRS2^−/−^ mice showed a similar activation of ACC than WT mice together with augmented FAS levels and fatty acyl chains, without changes in *Lpl* expression, suggesting that these changes are not due to elevated plasma uptake. FAS uses NADPH, and we found herein an increase in G6PD activity in ND IRS2^−/−^ mice. However, D IRS2^−/−^ mice presented a reduction in ME activity. A decrease in G6PD and ME activities leads to increased oxidative stress and apoptosis [27] as we previously described in D IRS2^−/−^ mice [4].

SCD synthesizes MUFAs and our results denoted an increase in SCD and oleate levels; being SCD levels regulated by insulin [28]. Thus, the increase in AKT phosphorylation in ND IRS2^−/−^ mice [3] might explain the higher levels of SCD and oleate. Synthesis of PUFAs depends on the novo lipogenesis and intake of essential fatty acids. In ND IRS2^−/−^ mice, the increase in linolenic acid can explain the higher levels of DHA and ω3-fatty acids. The increase in linolenic acid in these mice cannot be attributed to food intake, as ND IRS2^−/−^ mice ate the same as the controls, but less than D IRS2^−/−^ mice [3]. In this regard, diabetic patients have lower plasma levels of linolenic acid that may be related to higher concentrations of lipid peroxides that alter its metabolism [29].

Most of the beneficial effects of PUFAs have been attributed to their anti-inflammatory properties and the attenuation of insulin resistance. Several PUFAs impair activation of c-Jun N terminal kinase (JNK) and nuclear factor kappa B (NFκB) [30] and neurogenic activity induced by PUFAs increases the number of proopiomelanocortin (POMC), but not neuropeptide Y (NPY) neurons [31]. Our previous findings demonstrated an increase in JNK and NFκB activation, as well as an augment in NPY and a reduction in POMC expression only in D IRS2^−/−^ mice [3]. Oleate increases insulin signaling and reduces NPY expression [32] and linolenic acid may reduce insulin resistance through the increase in fatty acid oxidation in agreement with our data showing augmented *Cpt1a* expression in ND IRS2^−/−^ mice. Besides, linolenic acid and DHA are esterified into phospholipids that inhibit inflammatory responses and enhance insulin signaling [33].

One of the most striking findings was the pronounced inflammatory profile in D IRS2^−/−^ mice in the hypothalamus whereas ND IRS2^−/−^ mice presents an anti-inflammatory cytokine profile. This could indicate that central inflammation is likely one of the initial events that influence predisposition to diabetes in IRS2^−/−^ mice, leading to related-metabolic alterations [34]. Moreover, high fat diets trigger hypothalamic inflammation, often in the absence of changes in blood or white adipose tissue [35], suggesting that hypothalamic changes may be an early factor for development of metabolic disturbances. Anti-inflammatory cytokines such as IL-4 and -13 have the inverse effect, thereby activating AKT signaling pathway [36]. Nevertheless, we cannot discard that changes in glucose or other metabolites may modify the expression of inflammatory factors. Thus, MCP-1 is expressed in monocytes in response to a hyperglycemic milieu and monocyte-derived macrophages present in the arcuate nucleus may provoke metabolic disturbances [37].

Considering the polarization of macrophages, we found a similar classical activation in blood of ND and D IRS2^−/−^ mice, although according to their function, measured by circulating cytokine levels, D IRS2^−/−^ mice had a more adverse profile. In the hypothalamus, those mice showed a classical activation profile that is related to inflammation. The differential activation of the macrophage/microglia by different molecules (i.e., IFN-γ or IL-4) might trigger a response that depends on the source of the stimuli or receptor type in M1/M2 cells and the subsequent intracellular signaling [38]. The increase in the expression of the anti-inflammatory markers *Arg1* and *Mgl1* in ND IRS2^−/−^ mice and the reduction of *Mcr1* in D IRS2^−/−^ could be attributed to a different inflammation degree of astrocytes in ND and D IRS2^−/−^ mice [4].

We have reported increased oxidative stress, inflammation and insulin resistance in the hypothalamus of D IRS2^−/−^ mice [3,4]. Reduction of NAA in the brain is associated with diabetes comorbidities [39] since NAA is an anti-inflammatory molecule that controls the production of prostaglandins. Carnosine reduces the increase in proinflammatory cytokines in response to different stimuli, while an increase in carnosine degradation due to reactive metabolites of diabetes has been reported [40]. Additionally, niacinamide is relevant for the treatment of metabolic disorders and improves glucose metabolism [41].

The differential inflammatory and insulin signaling patterns in the hypothalamus of ND and D IRS2^−/−^ mice could be due not only to the differences in insulin levels/sensitivity, but also to additional factors. In this way, we have previously reported that phosphorylation of the IGF-I receptor was increased in the hypothalamus of ND IRS2^−/−^ mice, whereas oxidative stress was augmented in D IRS2^−/−^ mice [4] and these effects can contribute to the modulation of the inflammatory and signaling profiles in both groups of IRS2^−/−^ mice. Moreover, the increase in IRS1 levels and its association with p85 in ND IRS2^−/−^ mice, as well as the elevation of PTEN in D IRS2^−/−^ mice [3] can explain the differential activation of phosphatidylinositol-3 kinase pathway between ND and D IRS2^−/−^ mice.

A couple of caveats should be taken into consideration in this study. When we analyzed the effect of increased lipogenesis and unsaturated fatty acids on hypothalamic insulin sensitivity, we have to keep in mind that diets enriched in saturated fat may generate systemic insulin resistance, although the relationship between unsaturated-enriched fat diets and insulin resistance is controversial. Nevertheless, it is clear that high saturated fat intake has been associated with increased risk of glucose tolerance impairment [42], whereas diets with a high proportion of oleic acid can reduce liver steatosis and increase insulin sensitivity in experimental models of metabolic syndrome [43]. It is necessary to clarify not only the different types of fat in the diets, but also the lipid composition in the different organs and its relationship with the inflammatory environment and insulin sensitivity, as well as with hyperglycemia.

The second caveat is a possible activation of inflammation by hyperglycemia. Although in this model we have previously provided evidence that the development of diabetes from prediabetes is related to inflammation and changes in the activation of insulin signaling pathways in the hypothalamus [3], hyperglycemia may boost inflammation as reported [44]. Thus, differences in the glycemia of ND and D IRS2^−/−^ mice could explain the differential inflammatory milieu in the hypothalamus of both IRS2^−/−^ mice. More studies are needed to discern the mechanisms implicated in the changes of the inflammatory profile in these mice, which offer an excellent tool to analyze the factors that may differentially regulate hypothalamic insulin signaling leading to prediabetes or diabetes.

## 5. Conclusions

**Figure 6:**
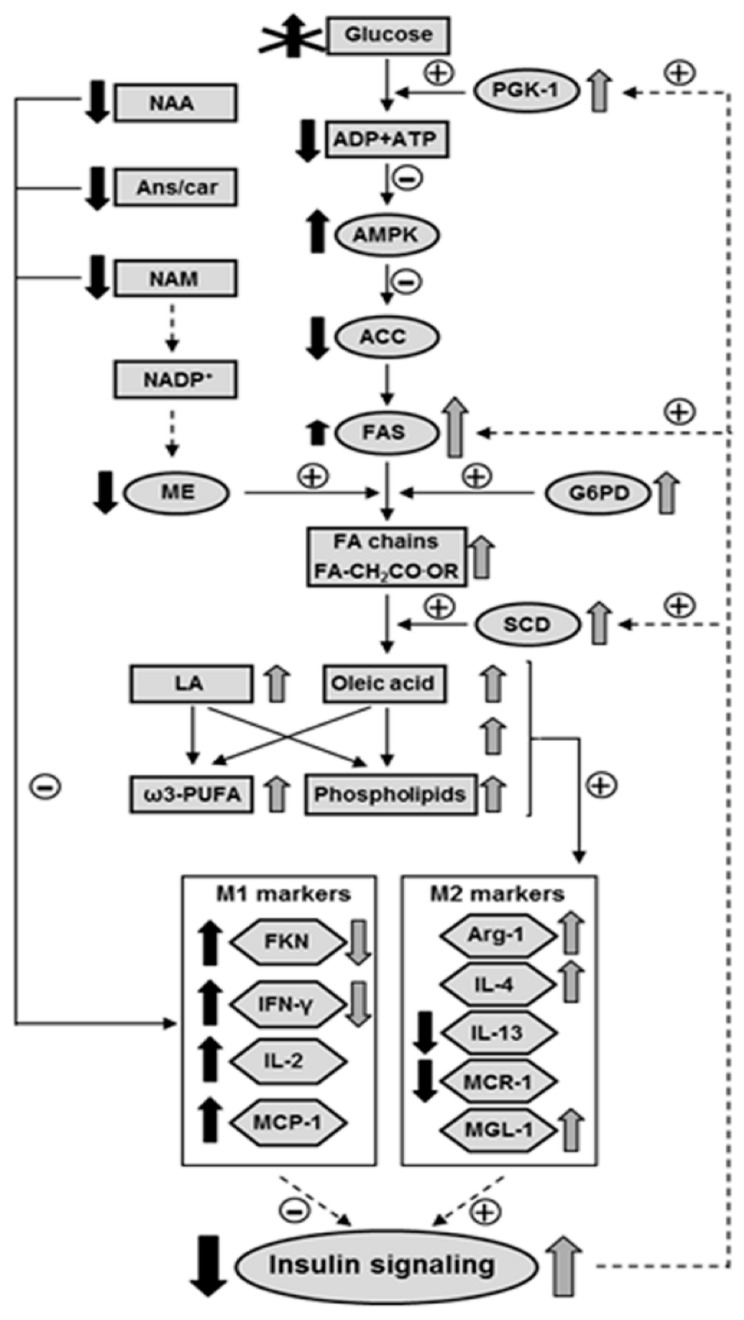
Proposed model for the interaction among inflammatory environment, insulin signaling and fatty acid profile in the hypothalamus of D IRS2^−/−^ and ND IRS2^−/−^ mice. Due to insulin resistance D IRS2^−/−^ mice do not use glucose properly. Decreased glycolysis reduced ATP levels, generating a negative energy balance that activates AMPK. AMPK inhibits ACC in D IRS2^−/−^ mice and enhanced insulin signaling up-regulates FAS in ND IRS2^−/−^ mice and, together with the NADPH generated by ME and G6PD in these mice, this may explain the higher levels of FA chains in ND IRS2^−/−^ mice. The increase in SCD levels, also regulated by insulin, generates monounsaturated fatty acids. There is also an increase in LA content, as well as in ω3-PUFA and phospholipids. All these changes may favor an anti-inflammatory environment in ND IRS2^−/−^ mice, that might increase insulin signaling in the hypothalamus. The decrease in anti-inflammatory molecules in D IRS2^−/−^ mice, such as Ans/Car, NAA or NAM, together with the absence of changes in PE, phospholipids and unsaturated fatty acids, could predispose to an increase in the inflammatory profile in these animals. Activation (or increase) and inhibition (or reduction) in D IRS2^−/−^ mice (black arrows) or ND IRS2^−/−^ mice (gray arrows) is indicated. Absence of arrows indicates the inexistence of differences with respect to WT mice. Length of the arrow shows relative degree of activation or inhibition. ACC, acetyl-CoA carboxylase; AMPK, AMP-activated kinase; Ans/Car, anserine/carnosine; FA, fatty acyl; FAS, fatty acid synthase; FKN, fractalkine; G6PD, glucose-6-phosphate dehydrogenase; IFN-γ, interferon-γ; IL, interleukin; LA, linolenic acid; MCP-1, monocyte chemoattractant protein-1; MCR-1, mannose receptor C-1; ME, malic enzyme; MGL-1, macrophage galactose-C type lectin-1; NAA, N-acetyl aspartate; NAM, nicotinamide; PE, phosphatidylethanolamine; PUFA, polyunsaturated fatty acid; SCD, stearoyl-CoA desaturase. Solid lines represent parameters studied here and dashed arrows symbolize previous results.

Our findings support the idea that the activation of lipogenesis and a subsequent increase in unsaturated fatty acid content in the hypothalamus of ND IRS2^−/−^ mice may counteract the inflammatory environment and, consequently, enhances the insulin signaling in order to facilitate energy supply for fatty acid anabolism. Conversely, the reduction in antioxidant and insulin-sensitizing markers in D IRS2^−/−^ mice worsens inflammation (Figure 6). These results suggest that an inadequate use of glucose in D IRS2^−/−^ mice reduces energy supplies in the hypothalamus, thereby activating AMPK which, in turn, inhibits lipogenesis. However, ND IRS2^−/−^ mice have an adequate energy status that favors hypothalamic lipogenesis, also modulated by insulin. In these mice, the increase in oleic acid and phospholipids, as well as polyunsaturated fatty acids, may promote an anti-inflammatory environment increasing insulin sensitivity and promoting the entry of glucose for anabolic processes. Future research will determine the relationship between changes in hypothalamic lipid metabolism and inflammation in the development of diabetes in this preclinical model.

## Figures and Tables

**Table 1:** Glycemia, HOMA-IR index and hormone and cytokine levels in serum and hypothalamus of WT, ND IRS2^−/−^ and D IRS2^−/−^ mice.

	WT	ND	D
Glycemia (mg/dL)	69 ± 3	159 ± 6 ***	596 ± 8 ***^###^
Insulin (ng/mL)	0.75 ± 0.07	1.90 ± 0.12 **	1.55 ± 0.13 **^#^
HOMA-IR	3.26 ± 0.37	18.59 ± 0.84 ***	57.07 ± 4.27 ***^###^
Leptin (ng/mL)	3.27 ± 0.23	7.79 ± 0.47 ***	0.17 ± 0.02 ***^###^
IGF-I (ng/mL)	149 ± 12	294 ± 21 ***	142 ± 10 ^###^
Serum IL-1β (pg/mL)	28.3 ± 4.8	43.9 ± 8.7	78.8 ± 10.4 *^#^
Hypothalamic IL-1β (ng/mg protein)	21.9 ± 2.8	12.1 ± 1.6 *	34.2 ± 3.4 **^##^
Serum IL-17	5.04 ± 0.56	7.97 ± 1.26	12.33 ± 1.85 **^#^
Hypothalamic IL-17 (ng/mg protein)	1.00 ± 0.14	1.02 ± 0.23	3.51 ± 0.26 ***^###^

Values are means ± SEM of six controls (WT), five non-diabetic (ND) and five diabetic (D) IRS2^−/−^ mice. HOMA-IR, homeostasis model assessment of insulin resistance; IGF-I, insulin-like growth factor I; IL, interleukin * *p* < 0.05, ** *p* < 0.01, *** *p* < 0.001 vs. WT; ^#^ *p* < 0.05, ^##^ *p* < 0.01, ^###^ *p* < 0.001 vs. ND. HOMA-IR index was calculated as glucose (mg/dL) x insulin (μU/mL)/405 as Heikkinen et al. [19].

## Data Availability

Not applicable.
